# First true blood pressure measurement with micropulse detection of arterial opening achieved

**DOI:** 10.1038/s41598-025-87855-2

**Published:** 2025-01-31

**Authors:** Alan Murray, Dingchang Zheng, Clive J. Griffiths, Chengyu Liu, David J. Graham, Jeff Neasham, Adrian J. Cossor

**Affiliations:** 1 Engineering School & Medical Faculty, Newcastle University, Newcastle upon Tyne, UK; 2 Centre for Intelligent Healthcare, Coventry University, Coventry, UK; 3 School of Instrument Science and Engineering, Southeast University, Nanjing, China; 4 A C Cossor & Son (Technology) Ltd, Anstey, UK

**Keywords:** Blood pressure measurement, Blood pressure accuracy, Blood pressure physiology, Calibration, International standards, Auscultation, Cardiology, Health care, Medical research, Engineering

## Abstract

There is a well-accepted need for a new more accurate solution to automatic blood pressure measurement, in spite of clinical convenience continuing to encourage the adoption of oscillometric devices with their known poor accuracy throughout healthcare. Our solution avoids estimates, mathematical modelling, oscillometric algorithms, and inadequate calibration against numerous private clinical data sets. Here we show that our new technique detects, from an arm cuff, micropulses associated with arterial opening between systolic and diastolic pressures; hence detecting systole at the first micropulse and diastole at the last, during cuff deflation. Our technique has equivalent accuracy to the gold standard auscultatory method, and is significantly better than the requirements of the current international standard for blood pressure devices. Our results provide scientific evidence for the effectiveness of our technique, and demonstrate significant clinically important improvements. We anticipate that our technique can be automated easily and economically. We acknowledge that this early study is an initial manual evaluation, but expect this new technique to be an automated universal solution for true blood pressure measurement, and a vital step change in an important clinical measurement in healthcare of the worldwide population.

## Introduction

### Deficiencies of current automated blood pressure devices

The Lancet published data on the global increase in the number of adults with raised blood pressure, which doubled over 40 years to over 1 billion people^1^. With the significant contribution of high blood pressure to cardiovascular disease, the World Health Organization^2^ introduced a global action plan to reduce the prevalence of raised blood pressure. To achieve this, accurate blood pressure measurement is essential.

Non-invasive measurement of blood pressure (BP) with a pressure cuff around the upper arm was introduced in 1896 by Riva-Rocci, using finger palpation of the physical pulse on the arm distal to the cuff to detect systole and diastole^3^. This was improved by Korotkoff, who detected sounds with a stethoscope over the brachial artery near the elbow during cuff deflation, as presented in 1905 in St. Petersburg^4^. This manual auscultatory approach, although needing much care that is not always given, continues to this day and is accepted as the “gold standard” for blood pressure measurement^5^, including with pregnant women and children^6,7^. However, the technique is not without its problems^6^.

In the 1980s, developers of automatic BP measurement used cuff pressure fluctuations induced by each heartbeat^8,9^. This approach is called the oscillometric technique and has been taken up by almost all automated device manufacturers. However, this technique has the significant disadvantage that each manufacturer uses their own proprietary technique, calibrated against their own private clinical auscultatory measurements. Nevertheless, the current use of automatic oscillometric blood pressure measurement far exceeds manual measurement. Despite their widespread use, it is acknowledged that oscillometric BP devices provide only an estimate of systolic and diastolic blood pressure (SBP and DBP), and require much needed improvements in accuracy^10^. As no suitable alternative is yet available, manufacturers are increasing production of existing devices, with estimated production to grow significantly^11^ and to be valued at US$7.98 billion by 2030^12^.

Stergiou et al.^13^ investigated what they called the “phenomenon of unreliable oscillometric BP measurement” and measured differences between simultaneous measurements by a professional oscillometric device and a mercury sphygmomanometer. Studying 755 patients (1706 clinical visits) they found errors greater than 10 mmHg in 15% of systolic and 6.4% of diastolic measurements.

The current guidelines from the European Society of Hypertension (ESH) acknowledge the potential for such measurement errors^7^. The Medicines and Healthcare products Regulatory Agency (MHRA), which is responsible for UK medical device regulation, recommends that “calibrated, non-mercury devices, which do not rely on oscillometry, are made available in all clinical areas”^14^, which currently includes calibrated non-mercury aneroid or electronic devices. Although there is reluctant acceptance that just an estimate of blood pressure is better than no measurement, there is wide agreement that a better technique is required^15^, where “greater collaboration between industry and academia would be an important first step”^16^.

### New blood pressure measurement technique using identification of micro pressure pulses associated with arterial opening under the blood pressure cuff

We describe for the first time a new technique for measuring blood pressure. It achieves accuracy, is not based on estimation, and avoids the unwanted necessity to calibrate against private clinical measurement datasets. Our new technique detects micropulses associated with arterial opening for each beat between systole and diastole, and so readily determines systole and diastole from the calibrated cuff pressure.

It does not require the use of a stethoscope as with gold-standard auscultation, or use the oscillometric technique. For each beat it detects arterial opening by our micropulse technique when arterial opening is physiologically present, and so identifies only those beats between systole and diastole. When the cuff pressure is above systole, the artery under the cuff is always closed during any heartbeat, and below diastole the artery is always open during any beat. When the cuff pressure lies between systole and diastole, the artery opens and closes during every beat, and this opening is detected by our technique. For cuff deflation, the first such beat identifies SBP and the last identifies DBP (and vice versa for cuff inflation). In addition, the identification of a continuous sequence of arterial opening for all beats between systole and diastole gives confidence in the results.

The relevant features of our innovation are described in our published patent by the research team Murray, Zheng, Griffiths and Liu^17^. Briefly, a pressure cuff is wrapped round the upper arm, as in any standard blood pressure measurement. The cuff has two pneumatic tubes, one for cuff inflation/deflation and also for determining the low-frequency cuff pressure and low-frequency heartbeat pressure changes, and the other tube for sensing the important high-frequency very low amplitude pressure changes that include the micropulses. The arterial opening events between systole and diastole are transient events, with high frequency components that are used to detect them.

Having first validated the principle, the approach is open for future automation, using techniques described in the patent^17^. Timing the interval between arterial opening and the start of the low-frequency arterial pulse shows that this interval shortens from one beat to the next during cuff deflation. As this will normally change smoothly during BP measurement, the sequence can be analysed for smooth beat-to-beat changes, providing reassurance for the measurement. For cuff deflation, the timing falls from about 200 ms to 50 ms; the actual values depending on heart rate and the patient’s cardiac condition. Systole is always detected near the peak of the low-frequency arterial pulse, and diastole close to the rising start of the pulse, and so earlier on the arterial pulse than for systole. The confirmation of this gradual change in timing gives added confidence in the measurement. Any irregular timing sequence can be identified, and clinicians could be requested to repeat measurements. However, in this research study all measurements were retained, and none was repeated for this reason. An added advantage is that any asystolic pause can be identified as a beat without arterial opening.

### Objective of research paper

This research paper presents an initial study evaluating the principle underlying our new micropulse technique for determining systolic and diastolic blood pressures. Results are compared with gold standard auscultation. We anticipate that our successful results will subsequently lead to full automation, and reliable accurate measurement of true BP.

## Methods

### Subjects and ethical declaration

32 healthy subjects were enrolled in this study. None had any known cardiovascular disease. The study received ethical permission from The Newcastle upon Tyne Hospitals NHS Foundation Trust Research Ethics Committee (REC number 11/NE/0340, CLRN ID 84514), and all subjects gave signed informed consent. The investigation conformed with the principles in the Declaration of Helsinki. For one subject the pressure sensors became accidentally misconnected during the recording, and in one other subject the cuff fastening was slipping during the whole cuff measurement making it impossible to analyse the stethoscope recording, giving 30 subjects for the study. Of the 30 subjects, 17 were male and 13 female, with an age range of 24–68 years (mean ± standard deviation (SD) 40 ± 12 years).

### Overview of methods

**Fig. 1:**
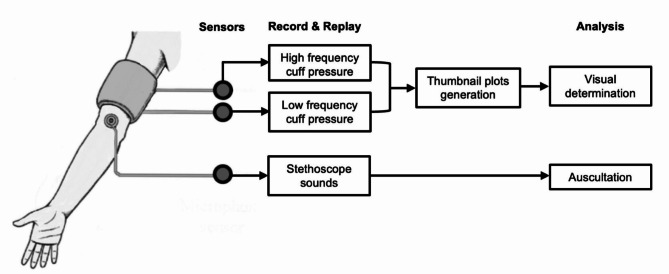
An outline of the main features of the research procedure showing where the signals originate, and which are stored for subsequent replay and human observer analysis.

Figure 1 provides an overview of the recording and replay method.

The figure illustrates the sensor detection of the low-frequency pressure signal, the micropulse pressure signal and stethoscope auscultation signal, followed by their recording and replay. The auscultation sounds are subsequently replayed via headphones. A numerical indication of cuff pressure in mmHg is also provided during auscultation. The filtered micropulse recordings are plotted for visual analysis as described below. The same cuff was used at the same time for both micropulse and auscultation recordings. This evaluation was during deflation only, but the technique works for both.

### Subject recordings

All subject data were recorded twice, the second time after a delay of 4 min. The arm cuff was not reapplied unless there appeared to be a problem, such as the cuff slipping. There were 2 pairs of measurements for micropulses and reference auscultation blood pressures for each individual recording, with 60 comparisons. Cuff inflation and deflation were automated. One appropriate cuff was selected from two cuffs. The Accoson Adult Cuff (bladder size 23 × 13 cm, arm range 24–34 cm) was used for almost all recordings. The Accoson Alternative Adult was used for the few recordings in the arm range 34–42 cm.

#### Recording procedure

All BP recordings were performed in a quiet clinical measurement room. Prior to the measurement, each subject was asked to rest on a chair for 5 min and to breathe gently during the whole measurement. The stethoscope head (bell side) was located downstream from the cuff over the brachial artery in the antecubital fossa with gentle skin contact pressure applied by a medical bandage. The whole measurement procedure followed the guidelines of the American Heart Association^18^. During each BP measurement the cuff was inflated, and then deflated linearly at the recommended rate of 2 to 3 mmHg/s.^19^ Since SBP is the first and DBP the last (during cuff deflation – or vice versa if inflation was to be used), and these pressures are associated with specific cuff pressures, the cuff pressure is the only calibration required. This is simply calibrated against a reference pressure, or with a pre-calibrated reference sensor. During cuff deflation, the low and high frequency cuff pressure signals and the stethoscope sounds were recorded with a sampling rate of 2 kHz and a resolution of 16 bits. The digitised signals were stored in a computer for subsequent off-line analysis.

#### Cuff pressure and micropulse recordings

A standard blood pressure cuff with two pneumatic port tubes was attached to the subject’s upper left arm. The cuff tubes were connected to the sensors as shown above. The low frequency port and sensor provided cuff pressure and heartbeat pulses, and the high frequency port and sensor, the micropulses.

#### Preparation of recordings for analysis

The low frequency cuff pressure recordings were filtered 0–15 Hz, and used to identify individual beats. The micropulse recordings were digitally filtered using a 4th order infinite impulse response (IIR) filter with a 3dB passband from 30 to 300 Hz. This filter mimics an analogue circuit response that was used in our initial research^17^.

### Analysis of stethoscope auscultatory sounds to obtain reference blood pressures

The stethoscope sounds were analysed independently by two experienced observers who had successfully completed the British and Irish Hypertension Society auscultatory measurement training [https://bihsoc.org/resources/bp-measurement/bp-measurement-auscultatory-tutorials/]. Both observers used the same computer replay software and the same high-quality headphones (Sony MDR-ZX100). Analysis of all recordings was repeated at two separate sessions with a delay to ensure that there was no memory of the first analysis. Observers were blinded to the results of the other observer and to their own first results. No recording was excluded in this evaluation, even when noise was present.

### Analysis of micropulse recordings

**Fig. 2:**
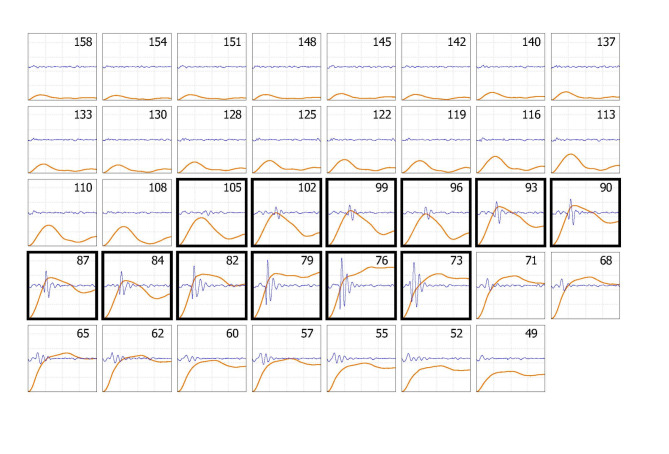
Example of a micropulse plot, containing all beats during deflation, shown in a thumbnail format. The only processing for each thumbnail is filtering. The cuff pressure for each is given in mmHg at the top right of the thumbnail, from 158 to 49 mmHg in this example. The thumbnails with a darker edge are those identified by the observers as having micropulses and lying between SBP and DBP. In this example the observers selected different beats for SBP with a one-beat difference, at 105 and 102 mmHg. They agreed for DBP at 73 mmHg. Observers gave identical results for the repeat analysis. The low frequency pressure pulses are shown in orange and the micropulse pressure signal in blue. The x-axis time scale is 0 to 400 ms from the pulse start. The y-axis scale is chosen to keep a constant range for all thumbnails for each subject, adjusted to suit the maximum amplitudes for the subject

For each individual, recordings of the micropulses were plotted in a series of beat-by-beat thumbnail plots. An example is shown in Fig. 2. Each thumbnail plot contained the micropulse and superimposed low frequency pulse. Both observers determined SBP and DBP visually and independently for each plot. This was achieved by identifying micropulses between SBP and DBP, as shown in the example figure. During cuff deflation, the first micropulse detected is at SBP and the last at DBP.

The observers confirmed that there was an expected clear transition at SBP, with continuing detection of micropulses until DBP is reached. These have a sharp leading edge, with some smoothing close to SBP, before full blood flow through the artery can be established. Immediately below DBP, a sudden waveform change is observed. Underlying remnants of large low-frequency arterial pulses are disregarded, as they are associated with the artery being always open below DBP.

Between SBP and DBP there is a smooth beat-to-beat transition in micropulse timing relative to the start of the low-frequency pressure pulse. At SBP the micropulse time is around 200 ms, usually with a low amplitude, and with an absence of similar pulses at higher pressures. With continuing deflation, micropulses increase in amplitude, becoming more complex in shape, and with an expected decrease in timing for each beat.

As for the analysis of auscultatory sounds, analysis of all recordings was repeated at a separate analysis session with a delay to ensure that there was no memory of the first analysis.

The example in Fig. 2 is shown because the two observers determined SBP with a one beat difference, at 105 and 102 mmHg. The micropulse at 105 mmHg is only just apparent, and this may be because the artery has only started to open before it then closes. Both observers determined DBP at 73 mmHg.

### Comparison of auscultatory and micropulse cuff measurements

The repeat analysis of auscultatory listening was used to determine operator variability, to ensure that the operator uncertainty did not contribute significantly to the overall results. In addition, as BP might be expected to fall a little during the interval between repeat measurements, changes in BP from the 1st to 2nd recording were analysed to determine if any change detected by auscultation was also detected by our new technique.

Finally, the blood pressures measured for all subjects with this new technique were compared with the “gold standard” reference auscultation measurements. The mean and SD of paired differences were calculated, and ‘Bland-Altman’ analysis and plots generated to provide overall comparative data^20,21^. In addition, the percentage of measurements agreeing with the “gold standard” auscultation measurements, within ranges of clinically accepted limits, was determined.

### Statistics

All statistical tests used two-tailed distributions, and all used an exact N of 30, for the 30 subjects studied. Paired t tests were used for all comparative differences.

## Results

### Repeat observer consistency for auscultation reference BP

The overall auscultation mean for the 30 subjects was 109.3 mmHg for SBP and 72.3 mmHg for DBP. The mean auscultation results from both observers were taken as the “gold standard” reference values for each subject. The difference between operators was not significant for SBP, although for DBP there was a small significant bias with one operator reading slightly higher than the other (1.01 ± 1.64 mmHg, t = 3.30, *P* = 0.003).

### Repeat observer consistency for the micropulse technique

The overall micropulse BP mean for the 30 subjects was 107.9 mmHg for SBP and 70.2 mmHg for DBP. The difference between operators was not significant for SBP or DBP.

### Differences between two sequential measurements for both techniques

An anticipated potential small change in blood pressure over the period between the sequential measurements was detected as a small fall in blood pressure with the new micropulse technique. These results were for SBP (-2.90 ± 5.13 mmHg, t = 3.04, *P* = 0.005), and DBP (− 1.43 ± 3.00 mmHg, t = 2.58, *P* = 0.032). Auscultation detected only a SBP change (− 0.28 ± 5.13 mmHg, t = 2.40, *P* = 0.023), with no change detected for DBP (− 0.78 ± 3.13 mmHg, t = 1.33, *P* = 0.19).

### Comparison of micropulse technique with auscultation reference

**Fig. 3:**
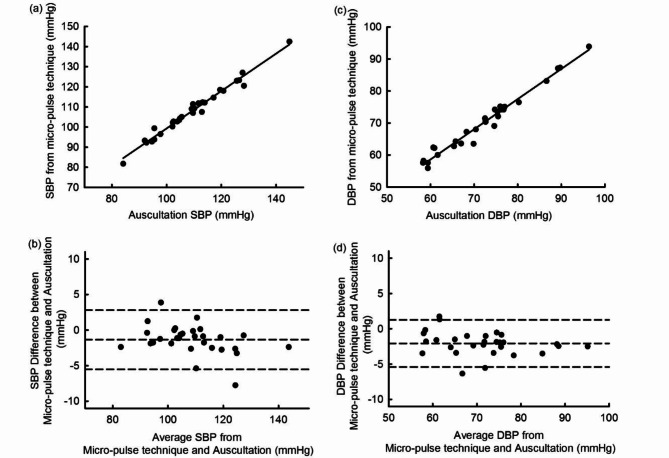
Correlation between the two techniques and Bland-Altman analysis. Comparisons show data from each of the 30 subjects.

The correlation between the two techniques was 0.989 (*P* < 0.00001) and 0.986 (*P* < 0.00001) for SBP and DBP respectively. Figure 3 shows the correlation between auscultation and micropulse measurements.

The Bland-Altman analysis is also shown in Fig. 3. There was a 2 mmHg bias between the micropulse technique and auscultation for DBP (mean ± standard deviation − 2.07 ± 1.67 mmHg, t = 6.70, *P* < 0.00001), and a smaller bias for SBP (− 1.34 ± 2.08 mmHg, t = 3.48, *P* = 0.0016). These mean difference values were highly significantly different (better) in comparison with the ISO Standard of 5 mmHg (DPB − 2.93 ± 1.67 mmHg, t = 9.48, *P* < 0.00001; SBP − 3.66 ± 2.08 mmHg, t = 10.2, *P* < 0.00001). Also, the variance ratio (F) between the techniques was highly significantly different (better) in comparison with the ISO Standard requirement for a SD of ≤ 8 mmHg (DBP F = 22.9, *P* < 0.001, SBP F = 14.8, *P* < 0.001). For DBP, all observer measurements agreed within 4 mmHg, and 94% within 2 mmHg. For SBP, 93% (56/60) were within 4 mmHg, and 88% (53/60) within 2 mmHg. There were 4 SBP measurements at or above 5 mmHg, which might have been identified as noisy with automation and so repeated.

## Discussion

We have shown that the micropulse technique achieves better results than required by the ISO Standard^6^ for automated blood pressure devices. There was no significant mean bias value for SBP, and the small 2.1 mmHg bias for DBP was well within the 5 mmHg limits. The SD of 2.1 mmHg for SBP and 1.7 mm Hg for DBP were much better than the 8 mmHg of the Standard. These results give encouragement to pursue full automation of the micropulse technique.

A small difference of less than 2 mmHg in DBP auscultation between observers was noted, perhaps due to small differences in hearing, despite both observers passing the British and Irish Hypertension Society’s auscultatory measurement training. The micropulse technique, once automated, will avoid the need for auscultation with any potential human error.

The concept underlying the principle of this unique technique should be straightforward to implement in an automated device. This would also bring with it opportunities for additional features, some of which are suggested below.

Of the two subjects excluded, one has continuous movement and noise from the fastener. This noise also influenced the analysis of other recordings that were not excluded as measurements could be made. Any subsequently automated device could detect such noise, and request or implement a repeat measurement, and so improve the measurements.

If the artery only briefly opens at systole, a very small micropulse is generated, which was the case in Fig. 2, and in which there was a 3 mmHg difference between observers, but this created only a single beat measurement difference.

An additional advantage of the technique is that it shows the expected change in timing between the start of the arterial pulse and the arrival of arterial opening, and shows opening for all beats between systole and diastole, giving confidence in the technique. This timing could easily be measured and displayed in any future automated device, providing reassurance to users.

This is a new, original and much needed step change in blood pressure measurement. This new technique overcomes the published “phenomenon of unreliable oscillometric blood pressure measurement”^13^. There is no need for calibration against reference subjects, as is required by manufacturers of current devices. The technique will also detect asystolic pauses, and has the potential for use in pregnant women and children, as all subjects whatever their condition have a physiological requirement for arterial opening below the cuff between SBP and DBP.

Our unique technique gives true reliable BP measurements rather than estimates, leading to the important and necessary improvement in clinical diagnosis and patient treatment.

Our micropulse technique now needs to be automated, and then evaluated with more subjects, with different clinical conditions.

## Data Availability

The data that support the findings of this study are available in: https://doi.org/10.25405/data.ncl.27263892.
